# Sex Differences in Insular Cortex Gyri Responses to the Valsalva Maneuver

**DOI:** 10.3389/fneur.2016.00087

**Published:** 2016-06-09

**Authors:** Paul M. Macey, Nicholas S. Rieken, Rajesh Kumar, Jennifer A. Ogren, Holly R. Middlekauff, Paula Wu, Mary A. Woo, Ronald M. Harper

**Affiliations:** ^1^UCLA School of Nursing, University of California at Los Angeles, Los Angeles, CA, USA; ^2^Brain Research Institute, University of California at Los Angeles, Los Angeles, CA, USA; ^3^Department of Anesthesiology, University of California at Los Angeles, Los Angeles, CA, USA; ^4^Department of Radiological Sciences, University of California at Los Angeles, Los Angeles, CA, USA; ^5^Department of Neurobiology, University of California at Los Angeles, Los Angeles, CA, USA; ^6^Department of Medicine, Division of Cardiology, David Geffen School of Medicine, University of California at Los Angeles, Los Angeles, CA, USA; ^7^Yale School of Medicine, Yale University, New Haven, CT, USA

**Keywords:** autonomic, sympathetic, fMRI, cardiovascular, functional neuroanatomy

## Abstract

Sex differences in autonomic regulation may underlie cardiovascular disease variations between females and males. One key autonomic brain region is the insular cortex, which typically consists of five main gyri in each hemisphere, and shows a topographical organization of autonomic function across those gyri. The present study aims to identify possible sex differences in organization of autonomic function in the insula. We studied brain functional magnetic resonance imaging (fMRI) responses to a series of four 18-s Valsalva maneuvers in 22 healthy females (age ± SD: 50.0 ± 7.9 years) and 36 healthy males (45.3 ± 9.2 years). Comparisons of heart rate (HR) and fMRI signals were performed with repeated measures ANOVA (threshold *P* < 0.05 for all findings). All subjects achieved the target 30 mmHg expiratory pressure for all challenges. Typical HR responses were elicited by the maneuver, including HR increases from ~4 s into the strain period (Phase II) and rapid declines to below baseline 5–10 s, following strain release (Phase IV). Small, but significant, sex differences in HR percent change occurred during the sympathetic-dominant Phase II (female < male) and parasympathetic-dominant Phase IV (female > male, i.e., greater undershoot in males). The insular cortices showed similar patterns in all gyri, with greater signal decreases in males than females. Both sexes exhibited an anterior–posterior topographical organization of insular responses during Phase II, with anterior gyri showing higher responses than more posterior gyri. The exception was the right anterior-most gyrus in females, which had lower responses than the four other right gyri. Responses were lateralized, with right-sided dominance during Phase II in both sexes, except the right anterior-most gyrus in females, which showed lower responses than the left. The findings confirm the anterior and right-sided sympathetic dominance of the insula. Although sex differences were prominent in response magnitude, organization differences between males and females were limited to the right anterior-most gyrus, which showed a lower fMRI response in females vs. males (and vs. other gyri in females). The sex differences suggest a possible differing baseline state of brain physiology or tonic functional activity between females and males, especially in the right anterior-most gyrus.

## Introduction

Sex differences in cardiovascular and other diseases may be driven in part by variations in autonomic nervous system function; identifying the neural source of such differences may improve understanding of mechanisms and potential treatment options. While many sex differences in cardiovascular function vary by age, with substantial variations between pre- and post-menopausal women, there nevertheless remain distinct characteristics of females compared with males (1, 2). For example, males show a higher prevalence of hypertension than females (3–5), but prognoses for women after myocardial infarction are worse than for males (6, 7). Such clinical sex differences are associated with autonomic function variation, including reduced muscle sympathetic nerve activity (MSNA) in women (8, 9), lower resting heart rate (HR) in men, differing HR variability patterns (10), sex differences in catecholamine responses to central sympathetic inhibition (11), and lower baroreflex sensitivity in females [(12); for review, see Dart et al. (13)]. One possibility is that enhanced alterations in sympatho-adrenal activation in males compared with females reflect greater sympathetic nervous system control in females, which is protective against hypertension and other cardiovascular disease (14). Many neural structures associated with autonomic functions show sex differences in function or structure at cellular and anatomical scales (15, 16). Brain regions serving autonomic regulation are potential therapeutic targets for interventions, but further understanding of sex differences in autonomic function of such regions is required.

The insular cortex is one key autonomic regulatory structure located under the parietal operculum and within the lateral sulcus (17–20). The insula receives input from multiple cortical areas involved with emotional, cognitive, and sensorimotor functions and projects to autonomic outflow areas in the brainstem both directly and *via* the hypothalamus (21–24). Although the insular cortices may not be involved in tonic maintenance of blood pressure (25), stimulation, stroke, lesion, resection, and MRI studies in human and non-human models provide evidence which demonstrates that those cortical areas serve roles in autonomic regulation (20, 25–39). The gyri of the insula are organized in a ventrodorsal orientation (18). Functions are topographically organized, in both human and non-human primates, with autonomic regulation, in particular, showing differentiation over the major gyri (40–46). However, the topographic differentiation of autonomic function by sex is unclear. Sex differences in spatial organization of insular function could contribute to sex differences in physiologic autonomic regulation (47).

Altered functions could arise from differences in structure. Males show larger insulae, with on average more gyral folding than females (16, 48). Larger brain weight and volume in males are consistent macroscopic findings, although one accounted for by their larger body dimensions (15, 49–51). Distribution of gray and white matter brain tissue compartments shows sex differences when adjusted for individual brain size (51–57). A recent meta-analysis of raw mean brain volumes for typically developing females and males found that the right insular cortex, right anterior cingulate gyrus, and bilateral thalamus gray matter densities were greater in female subjects, whereas the left anterior and posterior cingulate gyri and the right and left amygdalae have greater gray matter volume in male subjects (58).

The topographic organization of autonomic functions in the insula can be evaluated with functional magnetic resonance imaging (fMRI) during physiological challenges. One such stimulus is the Valsalva maneuver (44, 59), a standard test of autonomic function involving a forced expiratory effort against a closed glottis, resulting in increased thoracic pressure (60–63). The physiologic responses to the Valsalva consist of four phases, with different recruitment of parasympathetic and sympathetic systems, and the task allows differentiation of various components of the autonomic response, with amplitude and timing patterns that might differentiate sex roles (64–66). Phase II is the strain period 3–5 s after initiation of the challenge and is characterized by strong sympathetic activation, indicated by an increasing HR (67, 68). Sympathetic withdrawal follows in the Phase IV recovery period 3–5 s after pressure release, characterized by a rapid HR fall to levels below baseline, followed by a gradual return over minutes to starting levels (69–71). Variations from the normal response occur in people with impaired autonomic regulation (72).

The objective was to assess female and male insular gyral organization of fMRI responses to the Valsalva maneuver in healthy adults. We evaluated both anterior–posterior organization within left and right insular cortices, and the laterality of equivalent gyri, since the right insula has been shown to be dominant during sympathetic phases of autonomic stimuli (44); animal and human models also show a preferentially sympathetic role for the right insula (73–76). Based on sex differences in central and peripheral autonomic function, we hypothesized that insular organization would differ between the sexes. Based on greater cell densities in females, we hypothesized males would show lower magnitudes of responses than females. Based on the lower metabolism in females in the anterior insula, we hypothesize that the short gyri would show more substantial sex differences compared with the long gyri.

## Materials and Methods

### Subjects

We studied 57 healthy adults (age ± SD: 47.0 ± 9.1 years, range: 31–66 years; 37 males, 20 females). All subjects had no history of cerebrovascular disease, myocardial infarction, heart failure, neurological disorders, or mental illness and were not taking cardiovascular or psychotropic medications. Subjects were recruited from the Los Angeles area and did not weigh more than 125 kg or have any metallic or electronic implants; the latter two issues are MRI scanner contraindications. All subjects provided written informed consent in accordance with the Declaration of Helsinki, and the research protocol was approved by the Institutional Review Board of UCLA. No subjects were taking exogenous sex hormones (for example, oral contraceptive pills, hormone replacement therapy, or testosterone therapy).

### Valsalva Maneuver

The Valsalva maneuver was performed in a sequence of four 18-s exhalations against a closed glottis, spaced 1 min apart, to a target expiratory pressure of 30 mmHg (77). While 40 mmHg for 20 s is commonly used, we selected slightly lower pressure and duration to help ensure the task could be successfully performed by a wide range of subjects of differing age and health. A light signal was used to indicate onset of the challenge for the Valsalva effort to the subject. Subjects were instructed to, upon seeing the light signal, take a breath and exhale against a resistance, maintaining a target pressure. A second light was illuminated when the subject achieved this 30 mmHg target pressure. Subjects practiced the Valsalva maneuver prior to scanning, and the research team observed each challenge to ensure the target pressure was reached and maintained for each of the four expiratory periods.

### Physiologic Signals

Cardiac, load pressure and indicator signals (e.g., light on/off) were recorded with an analog-to-digital acquisition system (instruNet INET-100B, GWI Instruments, Inc., Somerville, MA, USA). HR was assessed using an MRI-compatible pulse oximeter (Nonin Medical Inc., Plymouth, MN, USA). The sensor was placed on the right index finger throughout the scan, and HR was calculated from the raw oximetry signal acquired at 1 kHz using custom peak-detection software followed by expert review. Expiratory pressure was measured *via* tubing connected to a pressure sensor (Omega Engineering Inc., Stamford, CT, USA) outside the scanner. Patient cue signals were simultaneously recorded, and all signals were synchronized to the MRI scans and data corresponding to the fMRI recording period extracted.

### MRI Scanning

Functional MRI scans were acquired using a 3.0-T scanner (Siemens Magneton Tim-Trio, Erlangen, Germany), while subjects lay supine. A foam pad was placed on either side of the head to minimize movement. We collected whole-brain images with the blood oxygen-level-dependent (BOLD) contrast [repetition time (TR)= 2000 ms; echo time (TE) = 30 ms; flip angle = 90°; matrix size = 64 × 64; field-of-view = 220 mm × 220 mm; slice thickness = 4.5 mm]. The spatial resolution was based on achieving whole-brain coverage with the fastest possible acquisition time. Two high-resolution, T1-weighted anatomical images were also acquired with a magnetization prepared rapid acquisition gradient echo sequence (TR = 2200 ms; TE = 2.2 ms; inversion time = 900 ms; flip angle = 9°; matrix size = 256 × 256; field-of-view = 230 mm × 230 mm; slice thickness = 1.0 mm). Field map data consisting of phase and magnitude images were collected to allow for correction of distortions due to field inhomogeneities.

### MRI Data Preprocessing

All anatomical scans were inspected to ensure the absence of visible pathology. For each fMRI series, the global signal was calculated and the images realigned to account for head motion. Subjects with large changes in global BOLD signal, or who moved more than 4 mm in any direction were not included in the study. Each fMRI series was linearly detrended to account for signal drift (but not global effects) (78) and corrected for field inhomogeneities, spatially normalized, and smoothed (8-mm Gaussian filter), and mean time trends from each voxel were calculated across all subjects, as well as the challenge-means across each of the four Valsalva periods. A mean image of all subjects’ spatially normalized, anatomic scans was created. Software used included the statistical parametric mapping package, SPM12 (Wellcome Department of Cognitive Neurology, UK; www.fil.ion.ucl.ac.uk/spm), MRIcron (79), and MATLAB-based custom software.

### Region of Interest Tracing

**Figure 1:**
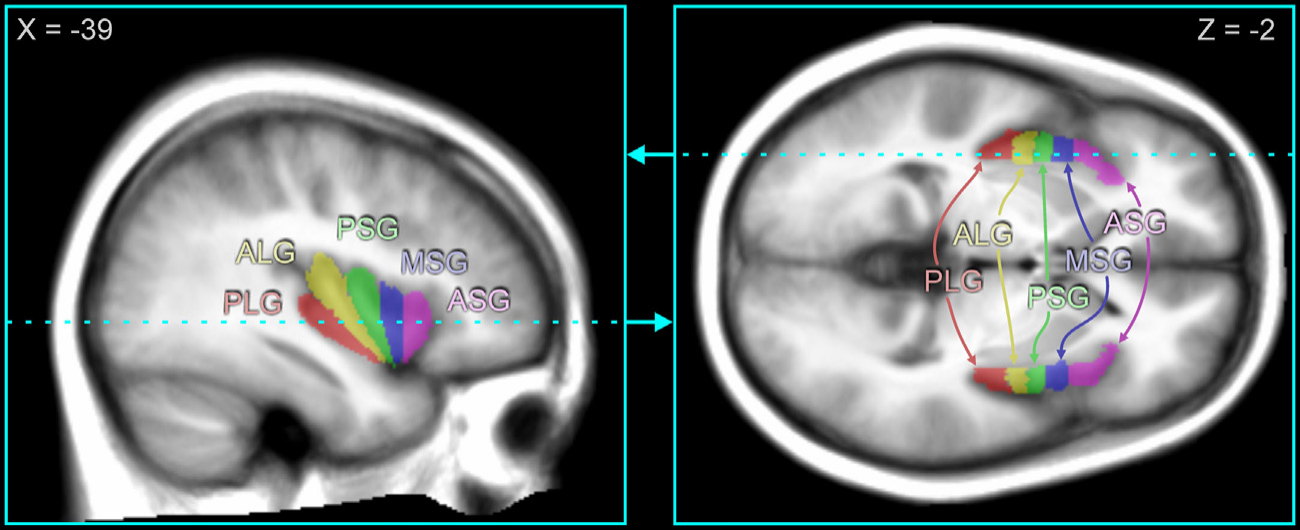
**Insular gyri color-coded and overlaid on average anatomical scan**. The anterior region of the insula is comprised of the short gyri, including the anterior short gyrus (ASG), mid short gyrus (MSG), and posterior short gyrus (PSG). The posterior region of the insula is comprised of the long gyri, including the anterior long gyrus (ALG) and posterior long gyrus (PLG). Coordinates in MNI space are indicated for each slice, and the slice location of the axial view is shown by the dashed line on the sagittal view and *vice versa*.

The five major gyral regions in the insular cortex, the anterior short gyrus (ASG), mid short gyrus (MSG), posterior short gyrus (PSG), anterior long gyrus (ALG), and posterior long gyrus (PLG), were outlined on the mean anatomical image with MRIcroN software (79), using previously published anatomical descriptions (18, 80). Figure 1 illustrates the gryi on an average anatomical scan in sagittal and axial views. While individual tracing would be more accurate for identifying the gyral differentiation on anatomical scans, the fMRI data are at a much lower spatial resolution (voxel volume of 53 vs. 0.8 mm^3^ for the anatomical scans), and the BOLD effect itself, which is the basis for assessing neuronal responses, is diffuse, so the advantage of individual tracing would be minimal. Since gyral folding in the insula has individual variation (48), the present approach distinguishes gyral regions rather than gyri *per se*. The three main gyri of the anterior insula, the ASG, MSG, and PSG, make up the convex surface of the structure and are visible on the sagittal and axial views of the mean anatomical image. The accessory and transverse gyri, two other gyri in the anterior insula, are difficult to visualize (80) and were not visible on the mean anatomical image. Thus, in our tracing of the ASG, we included the entire most-anterior portion of the insula, which included the accessory and transverse gyri. The posterior gyri (ALG and PLG) were easily visible on sagittal and axial sections of the anatomical volume.

### Statistical Analysis

Repeated measures ANOVA (RMANOVA), implemented with the mixed linear model procedure “proc mixed” in SAS 9.4 software (81), was used to identify periods of significant response relative to baseline, during the Valsalva and subsequent recovery periods (82). We modeled the fMRI responses as a function of scan timepoints. Significance was first assessed at the global level; as per the Tukey–Fisher criterion for multiple comparisons, the timepoints of significant responses were identified only for significant models (*P* < 0.05). Three sets of models were created: (1) within and between-group analyses of fMRI signal change relative to baseline; (2) separate female and male between-group signal change relative to PLG, where group consistent of five gyri in one hemisphere (with six between-gryi comparisons each); and (3) separate female and male signal change of left relative to right gyrus. Statistical assessment of sex differences for (2) and (3) were not performed as those signals were relative to another gyrus, such that the signal in males was relative to a different reference than females, which would complicate interpretation of RMANOVA-identified female and male differences. To avoid potential confounds due to global vascular effects, we focused on relative changes between gyri. The restriction of only assessing differences, rather than absolute responses, results from the relative nature of the BOLD-based fMRI technique. To identify anterior–posterior organization, we assessed responses with respect to the PLG, as the posterior insula typically responds less than anterior regions in response to autonomic stimuli (83). To identify lateral organization, we assessed right-sided relative to left-sided responses for each gyrus (44). We did not include a hemisphere factor in any model since the aims were restricted to identifying gyral-specific differences. Since age is a potentially confounding factor, especially in females given the changes in autonomic function associated with menopausal status (84), we implemented each RMANOVA model both with and without age as a covariate.

## Results

### Subjects

Females were, on average, slightly older than males (mean age ± SD: female 50.0 ± 7.9 years; male 45.3 ± 9.2 years), although the difference was not significant (*P* = 0.06, independent samples *t*-test). Body mass index also did not differ significantly (female 24.0 ± 5.1 kg/m^2^, male 25.2 ± 2.7 kg/m^2^; group difference *P* = 0.24, independent samples *t*-test). Six females reported being left-handed, two ambidextrous, and 14 right-handed. Four males reported being left-handed, two ambidextrous, and 30 right-handed. The difference was not significant (*P* = 0.2, chi-squared).

### Physiology

**Figure 2:**
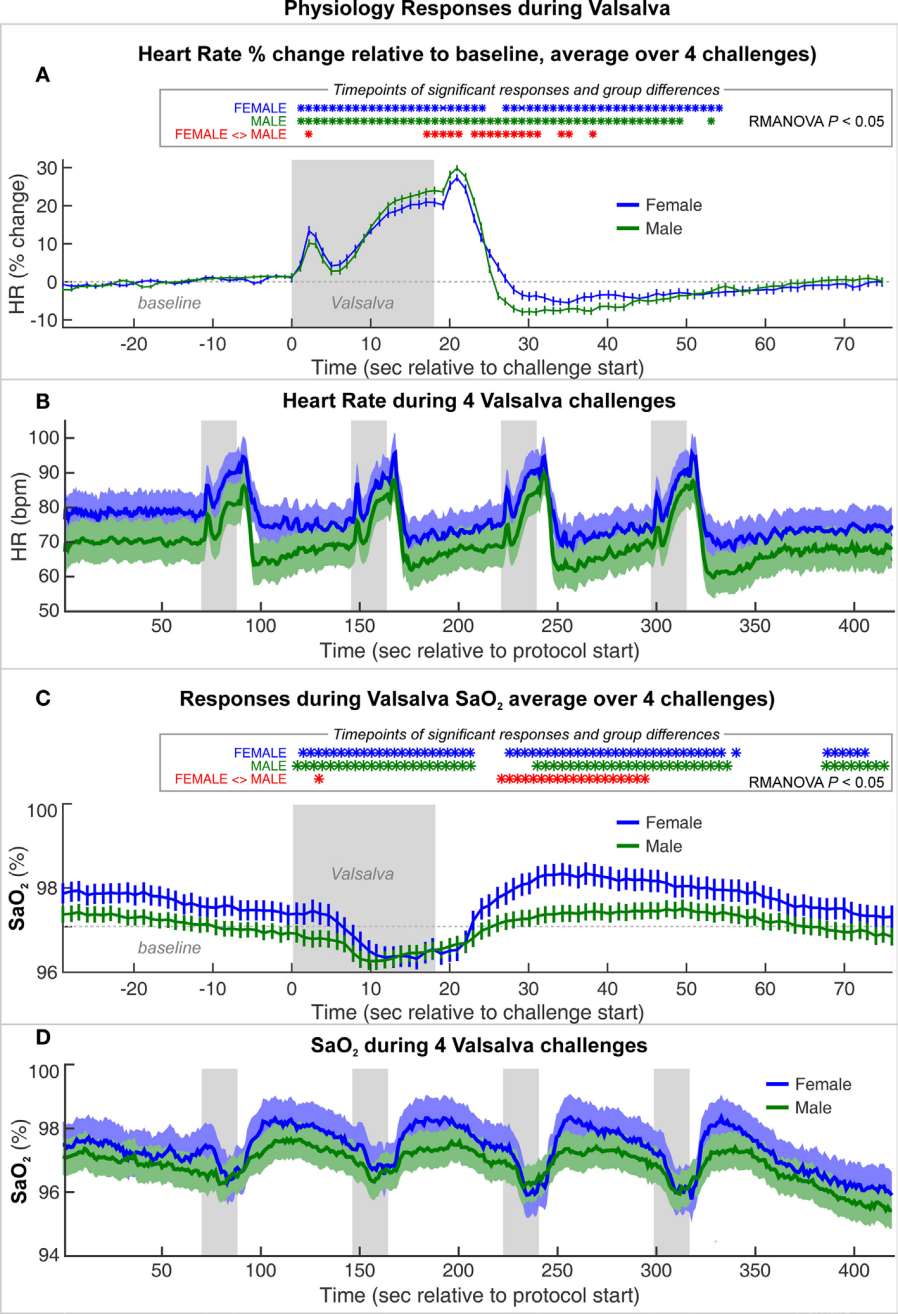
**Heart rate (HR) and SaO_2_ changes during a series of four Valsalva maneuvers, averaged for female and male groups**. **(A)** HR% change relative to baseline and **(C)** SaO_2_ averaged over four challenges (mean ± SE). Timepoints of significant within-group responses indicated in blue and green *, and significant group differences in red, based on *P* < 0.05 with repeated measures ANOVA (RMANOVA). **(B)** Absolute heart rate and **(D)** SaO_2_ over series of four Valsalva maneuvers, averaged over females and males with SE shaded.

The average HR changes in females and males illustrate the typical pattern of autonomic responses elicited by the Valsalva (Figure 2). The initial HR increase results from full inspiration prior to the forceful expiration, which leads to brief HR increases as compensation for the thoracic pressure changes, a period termed Phase 0 (85). While there were significant differences between females and males, notably smaller relative HR increases during Phase II and reduced undershoot in Phase IV, the magnitude of those differences was small, and both sexes showed the same pattern of responses (Figure 2A). The absolute levels show the expected higher HR in females over males (Figure 2B), and visual inspection of the sequence of four Valsalva challenges shows similar responses over the repeated maneuvers (Figure 2B).

Both groups showed a decrease in oxygen saturation (SaO_2_) from 10 s into the challenge, with a return toward baseline beginning ~8 s into the recovery period. The SaO_2_ was slightly higher in females than males during baseline and recovery periods (Figures 2C,D). However, females showed a greater decline during and after the challenge, such that the values were similar during the low SaO_2_ period. Female recovery was also larger, with an overshoot peaking ~20 s into the recovery period, whereas males showed a flat response.

### fMRI Responses

**Figure 3:**
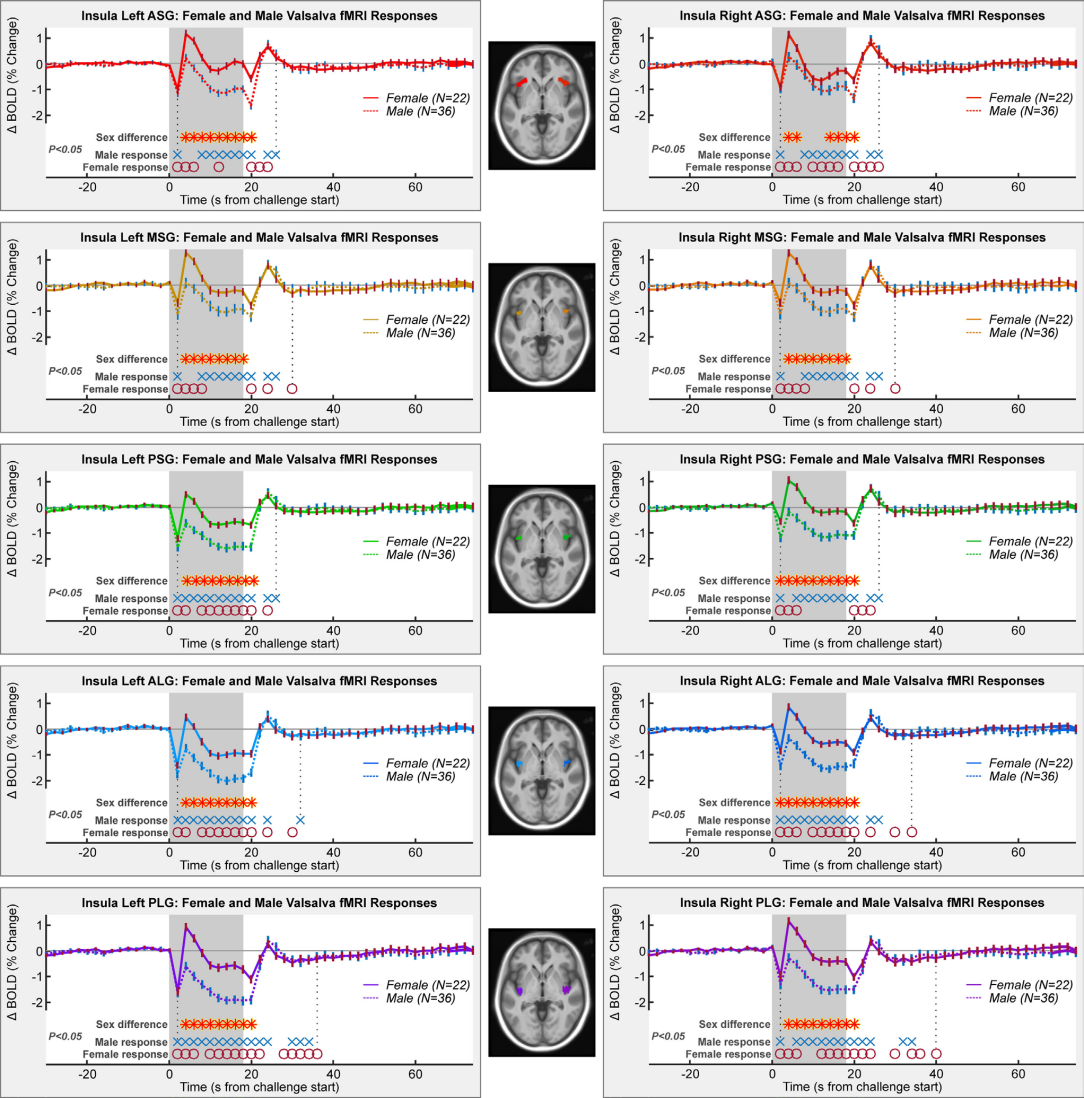
**Mean fMRI insula responses over four Valsalva maneuvers, averaged for female and male groups**. All left and right gyri patterns are shown. Timepoints of significant within-group responses and between-group differences are indicated above the *x*-axis and below the graphs (RMANOVA, *P* < 0.05).

The Valsalva maneuver elicited significant fMRI signal responses during the expiration period that differed from baseline in all gyri in both sexes (Figure 3). For both males and females, significant within-group responses were present throughout the strain period and during the recovery (blue X’s and red O’s in Figure 3).

Female and male responses differed in all gyri (Table 1; Figure 3). While the patterns included increases and decreases relative to baseline, the magnitude of female responses was consistently higher than males throughout the Valsalva period, as shown by the higher group averages (that is, the solid female lines higher than dashed male lines). Group differences were present from 4 s into the challenge, shortly after the start of the strain period (*P* < 0.05, RMANOVA red–yellow stars in Figure 3). The differences remained through to the end of the strain period, with the exception of the right ASG, where there were no differences from timepoints 8–12 s. The sex difference remained up to the first timepoint after the strain in the left ASG and in all posterior gyri (bilateral PSG, ALG, and PLG). The MSG showed no group difference during recovery.

**Table 1:** **Female vs. male model fit with and without age**.

Female vs. male	Group, time	Group, time, age	Group, time	Group, time, age
	Left ASG		Right ASG	
Model ChiSq (*P* < 0.0001)	454.28	456.57	600.52	603.27
−2 log likelihood	6925.1	6933.8	7856.9	7864.6
Group (*P*)	0.0028	0.0055	0.1888	0.3167
Time (*P*)	<0.0001	<0.0001	<0.0001	<0.0001
Group × time (*P*)	<0.0001	<0.0001	0.0066	0.0066
Age (*P*)		0.6743		0.2882
	Left MSG		Right MSG
Model ChiSq (*P* < 0.0001)	584.48	586.86	651.88	653.42
−2 log likelihood	6852.1	6860.5	8158.7	8166.1
Group (*P*)	0.0018	0.0045	0.067	0.1356
Time (*P*)	<0.0001	<0.0001	<0.0001	<0.0001
Group × time (*P*)	<0.0001	<0.0001	<0.0001	<0.0001
Age (*P*)		0.4375		0.2354
	Left PSG		Right PSG	
Model ChiSq (*P* < 0.0001)	647.74	648.73	583.37	584.68
−2 log likelihood	7070.9	7079.7	7519.7	7526.6
Group (*P*)	0.01	0.0121	0.0046	0.0175
Time (*P*)	<0.0001	<0.0001	<0.0001	<0.0001
Group × time (*P*)	<0.0001	<0.0001	<0.0001	<0.0001
Age (*P*)		0.8809		0.1001
	Left ALG		Right ALG	
Model ChiSq (*P* < 0.0001)	590.5	587.09	563.75	567.05
−2 log likelihood	7141.1	7149.4	7171	7178.4
Group (*P*)	0.0071	0.0065	0.0056	0.0175
Time (*P*)	<0.0001	<0.0001	<0.0001	<0.0001
Group × time (*P*)	<0.0001	<0.0001	<0.0001	<0.0001
Age (*P*)		0.5751		0.1769
	Left PLG		Right PLG	
Model ChiSq (*P* < 0.0001)	497.41	491.5	519.91	521.1
−2 log likelihood	7745	7753.2	7907.3	7915.6
Group (*P*)	0.001	0.0009	0.003	0.0064
Time (*P*)	<0.0001	<0.0001	<0.0001	<0.0001
Group × time (*P*)	<0.0001	<0.0001	<0.0001	<0.0001
Age (*P*)		0.4667		0.5565

*Overall model chi square (ChiSq) was always significant (*P* < 0.0001). The model fit is indicated by −2 × log likelihood as calculated by SAS (higher indicates better fit). The *P* values for each variable are shown; group × time and time were significant (*P* < 0.05) in all cases, and age was not significant in any model*.

### Anterior–Posterior Organization

**Figure 4:**
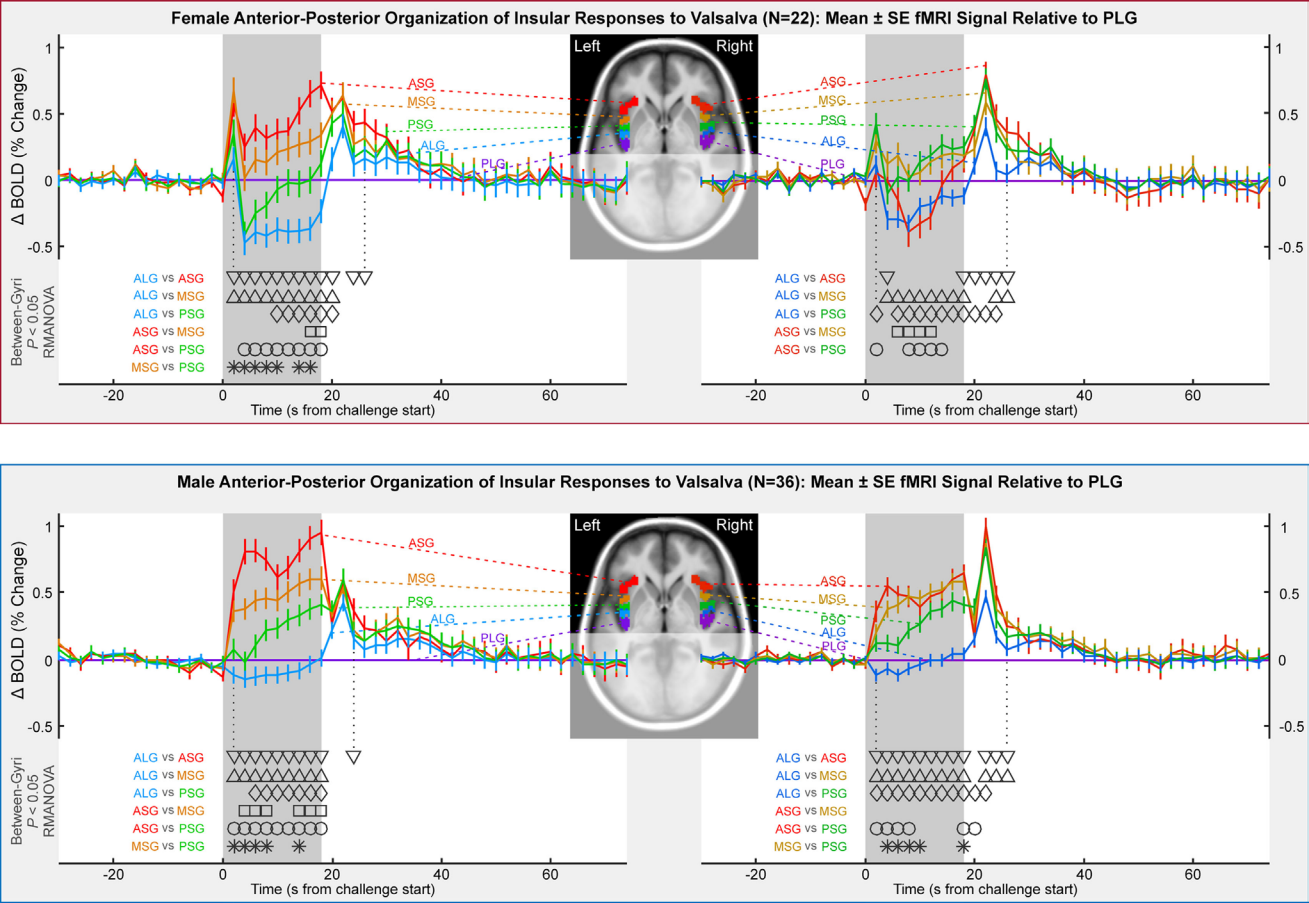
**Anterior-to-posterior organization of insula fMRI responses over four Valsalva maneuvers, illustrated by timetrends relative to pattern in posterior-most gyrus (PLG)**. Females in top and males in bottom. Timepoints of between-gyral differences are indicated by symbols above the *x*-axis and below the graphs (RMANOVA, *P* < 0.05).

Although most functional organization in terms of anterior–posterior and left–right relative responses was similar to the mixed group findings, differences appeared by sex, especially in the anterior insula (Table 2; Figure 4). An anterior–posterior organization was evident in the left insula, with ASG > MSG, MSG > PSG, and PSG > ALG. The organization of these four anterior-most gyri was similar in females and males, with significant differences between the regions during the expiratory period (*P* < 0.05, RMANOVA). However, the PLG in males showed a similar magnitude of response to the ALG, whereas in females, the PLG showed a magnitude between those of the MSG and PSG. In both sexes, the ASG demonstrated an elevated response relative to other gyri throughout most of the strain period (timepoints 4–18 s), with the MSG also showing a sustained, higher response relative to more posterior gyri. The same was true for the PSG relative to the ALG, and in males, the PSG relative to the PLG. In females, the PSG displayed significantly lower activity than the PLG for 6–10 s during the challenge. To summarize the left-sided patterns, males: ASG > MSG, MSG > PSG, PSG > PLG; females: ASG ≥ MSG, MSG > PSG, PSG ≤ PLG.

**Table 2:** **Anterior–posterior model fit with and without age**.

Anterior vs. posterior	Group, time	Group, time, age	Group, time	Group, time, age
	Female left		Female right	
Model ChiSq (*P* < 0.0001)	482.01	457.29	649.76	622.13
−2 log likelihood	5787.5	5793.2	4660.4	4665.9
Group (*P*)	0.0001	0.0001	0.0189	0.0153
Time (*P*)	<0.0001	<0.0001	<0.0001	<0.0001
Group × time (*P*)	<0.0001	<0.0001	<0.0001	<0.0001
Age (*P*)		0.0309		0.024
	Male left		Male right	
Model ChiSq (*P* < 0.0001)	1147.24	1113.27	1118.85	1110.22
−2 log likelihood	9826.7	9833.6	6499.1	6509.9
Group (*P*)	<0.0001	<0.0001	<0.0001	<0.0001
Time (*P*)	<0.0001	<0.0001	<0.0001	<0.0001
Group × time (*P*)	<0.0001	<0.0001	<0.0001	<0.0001
Age (*P*)		0.0486		0.3716

*Overall model chi square (ChiSq) was always significant (*P* < 0.0001). The model fit is indicated by −2 × log likelihood as calculated by SAS (higher indicates better fit). The *P* values for each variable are shown; group × time was significant (*P* < 0.05) in all cases, and age was significant for all models apart from male right*.

The right insula showed an anterior–posterior organization in males, although the ASG and MSG neither differ significantly nor did the ALG and PLG; the PSG responses were between these pairs of regions. In females, the right insular organization differed from that in males. The MSG and PSG were modestly higher than the PLG for half the strain period (6–14 s), whereas the ASG showed the lowest response in conjunction with the ALG. To summarize the right-sided patterns, females: MSG, PSG, PLG > ASG, ALG; males: ASG, MSG > PSG > ALG, PLG.

### Left–Right Organization

**Figure 5:**
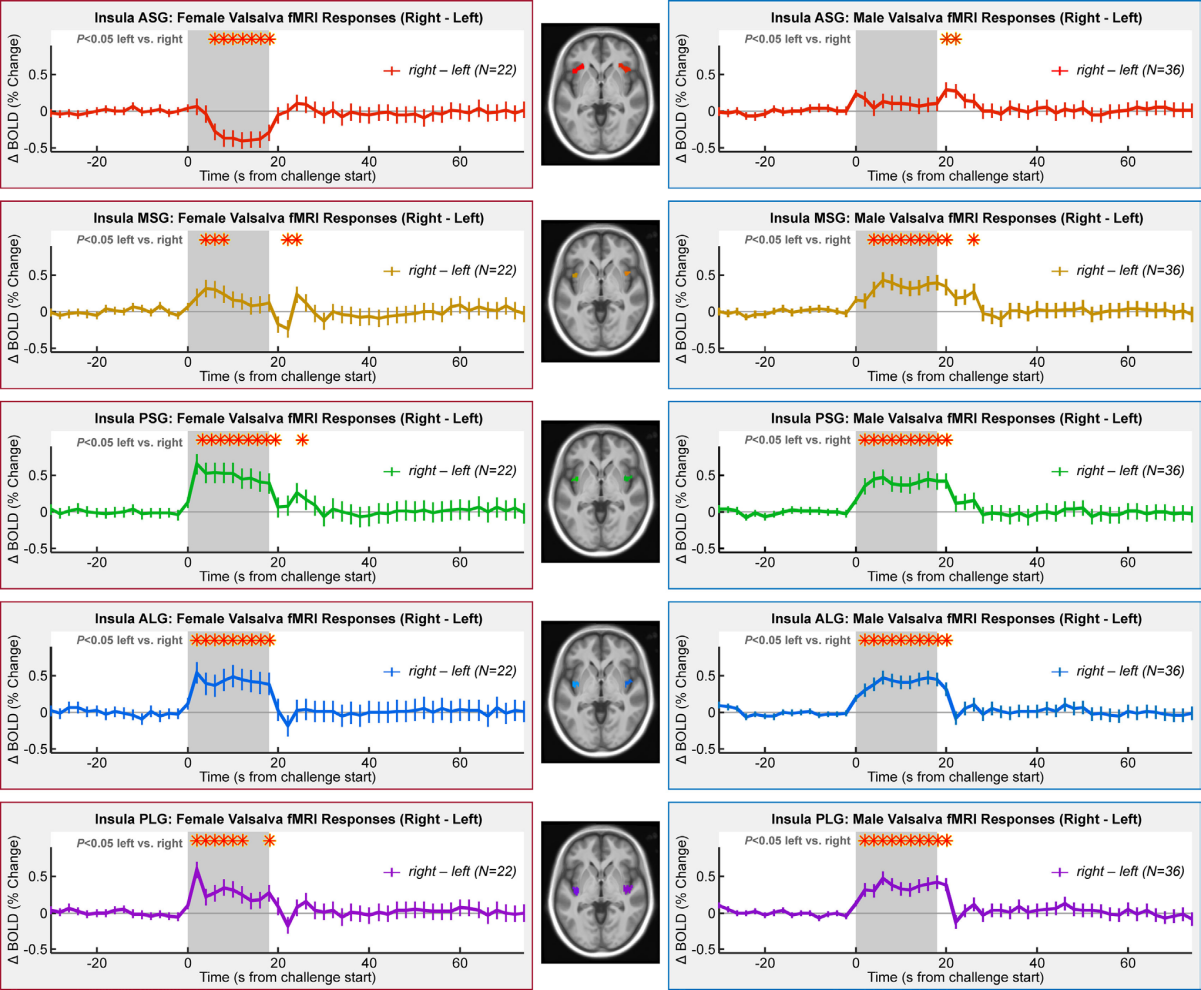
**Lateralization of insula fMRI responses over four Valsalva maneuvers, illustrated by right–left time trends, such that a higher signal indicates a greater right-sided response**. Females in left column and males in right column. Timepoints of between-hemisphere differences are indicated by a red * above the graphs (RMANOVA, *P* < 0.05).

Lateralization of activity during the challenge was evident in all gyri with the exception of the ASG in males and MSG in females (Table 3; Figure 5). However, the pattern of lateralization differed between sexes. Males showed greater activity in the right over left throughout the expiratory period in all gyri except the ASG. Females showed similar sustained greater activity in right vs. left in long gyri and PSG. In the MSG, there was no significant effect (Table 3). In the ASG, females showed the opposite pattern to males and other gyri, namely, a signal decrease in right vs. left during the expiratory phase.

**Table 3:** **Left vs. right model fit with and without age**.

Right vs. left vs. male	Group, time	Group, time, age	Group, time	Group, time, age
	Female ASG		Male ASG	
Model ChiSq (*P* < 0.0001)	168.98	157.17	427.43	421.36
−2 log likelihood	1725.5	1732.2	3132.8	3140.6
Time (*P*)	0.0002	0.0002	0.743	0.743
Age (*P*)		0.2305		0.4202
	Female MSG		Male MSG	
Model ChiSq (*P* < 0.0001)	145.47	137.54	267.25	248.68
−2 log likelihood	1822.5	1829.5	3516.3	3522.7
Time (*P*)	0.0875	0.0875	<0.0001	<0.0001
Age (*P*)		0.2834		0.1318
	Female PSG		Male PSG	
Model ChiSq (*P* < 0.0001)	196.2	161.88	207.28	186.97
−2 log likelihood	2152.7	2156.8	3706.4	3712.1
Time (*P*)	<0.0001	<0.0001	<0.0001	<0.0001
Age (*P*)		0.0621		0.0836
	Female ALG		Male ALG	
Model ChiSq (*P* < 0.0001)	213.22	191.21	223.1	224.94
−2 log likelihood	2287.4	2292.7	2964.9	2974
Time (*P*)	<0.0001	<0.0001	<0.0001	<0.0001
Age (*P*)		0.146		0.8333
	Female PLG		Male PLG	
Model ChiSq (*P* < 0.0001)	177.37	151.74	205.7	207.85
−2 log likelihood	1744.4	1749.7	3032.6	3041.8
Time (*P*)	0.0005	0.0005	<0.0001	<0.0001
Age (*P*)		0.0928		0.9854

*Overall model chi square (ChiSq) was always significant (*P* < 0.0001). The model fit is indicated by −2 × log likelihood as calculated by SAS (higher indicates better fit). The *P* values for each variable are shown; time was significant (*P* < 0.05) in all cases apart from male ASG and female MSG, and age was not significant in any model*.

### Age Influences

Inclusion of age in the models testing above did not alter the significance of group, time, or group by time variables (Tables 1–3). In all models, the inclusion of age slightly improved the model fit as indicated by higher chi square and −2 log likelihood values. As a variable in these models, age was only significant in anterior–posterior comparisons, on both sides in females and on the left only in males.

### Summary of Key Finding: Sex Differences in Right ASG

Comparison of ASG responses in Figures 3 and 4 show that male and female responses in the right ASG show the most substantial differences of any region, leading to both lateralization and anterior–posterior organization differences in the ASG region. These substantial sex differences in both magnitudes and patterns of fMRI signals occurred, despite only moderate variations in HR responses to the Valsalva maneuver.

We made available summary data including RMANOVA models and results, figures, and preprocessed fMRI images in a repository (86); the full raw dataset could not be provided to due the consent acquired at the time.

## Discussion

### Overview

Responses in both the right and left insular cortices during the strain phase of the Valsalva maneuver differed between sexes, with larger magnitude fMRI signal declines in males over females in all gyri. Similar anterior–posterior distributions of activity appeared in both males and females. Lateralization showed a right-sided dominance during the strain phase in all gyri in males, and in all but the anterior-most ASG in females, consistent with the right-sided sympathetic dominance previously reported (73). Thus, the most notable differences between sexes were the magnitude of response and the unique responses in females in the right ASG. These male–female differences in insular function may contribute to sex differences in cardiac responses to autonomic challenges and, over time, cardiovascular disease. The strain phase of the Valsalva is characterized by a large and rapid increase in sympathetic activity, as reflected in the HR increase; thus, the general findings corroborate the more anterior location of autonomic control within the insula.

### Insular Role in Regulation: fMRI and Physiology

Heart rate changes exhibited a typical pattern for healthy adults, with only minor sex differences, consistent with other findings (87). The fMRI time course in males and females followed an approximate inverse relationship with the HR changes, with brief insular deactivations at inspiration and beginning of expiration, declines during the sympathetic Phase II, a brief trough after release, followed by an overshoot and return to baseline in Phase IV (47). Oxygen saturation decreased in the latter half of Phase II and remained low for ~8 s following recovery, with a rebound in ~15 s into Phase IV; the oxygenation changes likely reflect lack of ventilation during breath-hold then higher ventilation after termination of the Valsalva. The fMRI signals did not appear to follow the oxygenation saturation patterns. While the fMRI changes could reflect a sensory role, with neural responses based on blood pressure and related afferent signals, human stroke and animal stimulation studies suggest the structure participates in regulation of autonomic functions (24, 74, 88–90). In particular, the observed pattern is consistent with the insula having an inhibitory role on HR changes. However, the insula does not show the exact opposite pattern to the HR changes since the functional role in cardiovascular regulation is more complex than a direct, proportional inhibition. The fMRI signals in all gyri return to baseline levels relatively early in Phase IV (which can last several minutes), suggesting the structure is not involved in sustaining sympathetic withdrawal. The insula has projections to the hypothalamus, a key autonomic region closely linked with medullary outflow areas (91, 92), as with other cortical areas, the insula shows inhibitory roles over lower brain regions (93). However, the male participants consistently showed greater insula declines in fMRI compared with females, even though HR changes were similar; in other words, the differences in magnitude of insula response did not lead to a difference in HR changes, at least as measured by percent change. Other challenges have been reported to also elicit sex differences in insular responses; lower body negative pressure leads to greater right, but not left insular activity in males compared with females (94), and a hand grip task leads to higher right-sided responses in males compared with females (95). The reasons for greater insula deactivation to the Valsalva maneuver in males are unclear, but could reflect a different baseline state or a different neural response. Cerebral blood flow (CBF) differs by sex, but in young adults, the insula shows lower metabolism in females compared with males (96).

### Lateralization

The lateralized organization of the insular responses in all but the anterior-most gyri showed greater right-side activation during Phase II of the Valsalva in both males and females, consistent with a preferentially sympathetic role of the right over left insula (73–76). The same patterns appeared in a combined female/male group (44). Similarly, left-sided insula injury is associated with greater parasympathetic than sympathetic impairment (97, 98). In some circumstances, the rat posterior insula shows no lateralization of cardiovascular function in response to stimulation (90); while differences between rat and humans (74) may extend to lateralization, these recent data, nevertheless, show that the insula has complex topographic organizational and different response patterns arise from different stimulation types. The two hemispheres also show strong connections in the anterior insula (99). Nevertheless, human stroke findings are consistent with a right-sided sympathetic regulatory role (28, 100, 101). Resection of the right insula due to the presence of a tumor also results in greater parasympathetic activity (38). Functional responses to a hand grip challenge also show right-sided dominance during sympathetic phases, for both left- and right-handed tasks (102). Further support is provided by a meta-analysis of neuroimaging findings (36). The present findings support a right-sided sympathetic dominance for at least some types of autonomic regulation, including, in this case, increased intrathoracic pressure.

### Anterior Sympathetic Dominance

During the sympathetic-dominant Phase II of the Valsalva, the anterior gyri showed greater fMRI responses compared with posterior gyri, with the exception of the ASG in female subjects. Other imaging studies show similar preferentially anterior insular roles in physiologic responses (103). At a cellular level, the rat insula shows varying structure and propagation properties in the rostral–caudal direction [equivalent of anterior–posterior in humans (23, 104)], consistent with varying functional topography. Anterior function is more closely associated with visceral reactivity in the rat (40). Some of the original evidence supporting lateralization of sympathetic and parasympathetic function was based on right anterior insula stimulation, showing HR increases in rats (74). Supporting these earlier animal findings, in a healthy adult human population (males and females combined), the Valsalva shows a distinct right-sided dominance during the sympathetic Phase II period (44). Sex differences in the anterior-dominant sympathetic roles are highlighted by lower body negative pressure studies, which elicit greater right anterior insular activity in males than females, together with different physiological responses (94); males also showed greater reductions in left anterior insular activity. The present findings, in all but the right ASG in females, are thus consistent with current understanding of anterior–posterior distribution of autonomic function.

### Sources of Sex Differences in Brain Function

A number of sex-based structural issues may contribute to the regional variation in functional responses found here. The middle short gyrus (MSG) in males is more distinct than in females; this sub-region was also more distinct in the left hemisphere, suggesting other sex and laterality variations (16). Females show higher gray matter volume in the right insular cortex than males (58). However, males show a greater number of distinct gyri on average [4.7 vs. 4.1 (48)]. Insular fibers also vary by sex; these fibers are influenced during development by hormonal status (105), specifically, connections between the left insula and left precentral gyrus show a lower density during development in females vs. males (106). In addition to structural differences, insular metabolism in young adults is lower in females, compared with males, but this difference is reduced in more posterior regions (96).

The global sex differences in functional responses could arise from varied sources, including metabolism, CBF, and hormone levels, as well as functional state. Sex hormones influence function (107–109) and have neuroprotective effects (110). CBF is higher in females than males, and activity-related CBF changes vary by sex (111, 112). Metabolism shows variation by sex in the parietal cortex and ventrolateral prefrontal cortices in adults (113), as well as the insula in adolescents (96). Brain functional state likely also differs between females and males in the insula; for example, both basal state and stress responses mediated by the hypothalamic–pituitary–adrenal axis show extensive sex variations (114). The sex differences in these physiologic variables likely lead to sex differences in baseline state and responsiveness of insula function.

### Differentiating the Right ASG

The anterior-most ASG showed patterns distinct from other female gyral responses as well as from male ASG responses. Since the HR changes were similar between sexes, this lateralization difference reflects either a differing baseline state or an altered organization. Considering the activation differences extend across all gyri, females and males may differ both by baseline state and by organization on the anterior short gyri. This altered organization of the anterior gyri was also reflected in within-structure, anterior-to-posterior organization, but with the differences appearing principally on the right side, with the anterior and mid short gyri showing relatively lower fMRI signals in females, in contrast with the higher activation in males. On the left side, both sexes demonstrate greater anterior gyral activation. The combined findings, therefore, suggest differing organization between females and males of the right ASG only, as opposed to differing baseline states.

### Clinical Implications

A range of behavioral and physiological characteristics are mediated by insular structural and functional properties. The sex-based variations in the structure may mediate mechanisms underlying significant health issues. The blood pressure regulatory roles served by the insula likely contribute to the higher lifetime risk of stroke in women over men, with that preference principally found in older women (115). Age-related findings may directly reflect developmental changes in neural maturation or later in neuropathology unique to the insula. Alterations of the insular cortices appear prominently in structural MRI studies of depression, and women are twice as likely to experience depression signs (116). The insula is frequently implicated in anxiety (117, 118), and women show more prevalent and more disabling anxiety disorders (119). The incidence of migraine is higher in females (120), and the insula serves a significant role in integration of pain signals (121). Thus, differences in the integrity of structure and function of the insula by sex should be considered in evaluation of central contributions to multiple medical disorders.

### The fMRI Signal and SaO_2_ Influences

The fMRI BOLD signal is influenced by baseline factors and changes in cerebral blood volume and metabolism and thus is not necessarily only a measure of neural activity. Sex differences in SaO_2_ included slightly higher baseline and recovery levels and a deeper fall and larger overshoot during recovery. Since fMRI signals are sensitive to baseline blood oxygenation, these differences in SaO_2_ could have influenced the findings (122). Conceivable, a higher SaO_2_ could lead to a ceiling effect for the females relative to males and thus limit the possible magnitude of signal change as seen in Figure 3; however, if all factors are equal, a higher baseline BOLD level would result in a larger signal change (122). The dynamic sex differences in SaO_2_ response are unlikely to have influenced the left–right or anterior–posterior differences since those effects occurred later in the challenge and lasted into the recovery period. Furthermore, global blood oxygenation levels as measured with a pulse oximeter would affect all regions similarly and thus should not contribute to region-specific variation. A final consideration is that challenges such as the Valsalva that elicit large changes in blood pressure lead to CBF and volume changes that dominate over oxygenation and neuronal activity influences, and variations in both baseline and responses of vascular state could have differed by sex. Such influences are by their nature global and could have influenced the consistent magnitude sex differences (Figure 3), but the left–right and anterior posterior differences (Figures 4 and 5) should principally reflect neuronal factors.

### Age Influences

There may be a modest confound of age since the sample was not exactly aged matched, with females being older albeit not significantly so (50.0 vs. 45.3 years, *P* = 0.06). Females in particular show changes in autonomic function associated with age and menopause (84, 123). However, although age impacts autonomic function, indices of the Valsalva maneuver, such as the Valsalva ratio, vary little or not at all with age (1, 65). If the sex differences were primarily due to age, the HR alterations should also show large sex differences. However, visual inspection of the magnitude of HR differences (Figure 2) relative to the fMRI differences (Figures 3–5) shows the cardiovascular responses differ only modestly, whereas the fMRI signals vary substantially, sometimes in opposite directions. The Valsalva challenge is therefore eliciting a similar albeit not equal peripheral response, but with substantial differences in neural response. Confirming the minimal influence of age, the inclusion of this variable in the RMANOVA models did not alter the patterns of difference in any model.

### Limitations

The sample included left-handed, right-handed, and ambidextrous subjects; since handedness is associated with variations in some autonomic measures, such as QRS-T interval (124) and MSNA responses, although not cardiovascular responses to hand grip (125, 126), this factor may have confounded the findings. Since the handedness distribution was similar between sexes, the effect is likely to be one of increasing variability and reducing sensitivity to differences (i.e., increase false negatives). Menopausal status menstrual cycle stage and hormonal contraceptive use were erroneously not assessed, but the sample likely includes pre-, peri-, and post-menopausal women. Since menopausal status and stage of the menstrual cycle influence many aspects of autonomic function (84, 123), the female measures may have been confounded by hormonal influences. Such effects would likely increase variability in the female group, which would reduce statistical sensitivity; thus, the sex differences reported here are unlikely to be false positives. We assessed topography distinguished by gyri, which run essentially anterior–posterior, but there is likely also functional differentiation across an approximate inferior–superior direction, given the cytoarchitectonic gradient in the insula from agranular cortex in the (inferior) anterior part to dysgranular cortex in the middle part to granular cortex in the posterior part (127–129). However, clear topographical distinctions based on MRI scans beyond gyri are likely to remain complex, given the heterogeneity of this structure (130–132). The short gyri, in particular, are not always distinct (16, 48); thus, the classifications ASG, MSG, and PSG should be considered to refer to adjacent regions that, in some subjects, are not distinguished by a gyral fold. Our focus in the present study was gyral-specific patterns, but variations in function or vascular responses across hemispheres could be assessed using similar models with the addition of hemisphere as a factor. Other limitations include the relative nature of fMRI signals such that only functional responses can be measured, but not baseline activity. Functional responses are measured as regional blood volume and oxygenation changes, which while strongly associated with neuronal activity (133), are also influenced by whole-brain cerebral vasculature. However, such global effects would not alter the between-gryi and lateralization organizational patterns identified in the current study.

## Conclusion

The insula shows sex differences in the topographic organization of functional responses during the sympathetic phase of the Valsalva maneuver. The response patterns are consistent with a principally inhibitory role for the structure. While the preferentially anterior autonomic function and preferentially righted sided sympathetic roles for the insula are shared in males and females, females show a smaller magnitude of change in fMRI signals than males in insular gyri. The exception is the right anterior-most gyrus (ASG) in females, which shows lower responses, compared with other female right and left anterior gyri, and compared with male gyri. The sex differences reflect altered baseline state and magnitude of responses and likely contribute to sex-related variations in autonomic responsiveness to some stimuli.

## Author Note

The mechanism of the tachycardia during the maneuver remains controversial, some workers suggesting exclusive enhancement of sympathetic activity and others suggesting depression of parasympathetic outflow; also, both parasympathetic withdrawal and increased sympathetic outflow have been suggested (Bennett et al., 1976; Eckberg, 1980). The quantification of heart interval variations [P65 (Junqueira, 2002)] impaired autonomic control of heart Valsalva.

## Author Contributions

Conception or design: RH, PM, and MW. Acquisition: RH, RK, PM, and MW. Analysis: PM, NR, and PW. Interpretation: RH, RK, HM, PM, JO, and NR. All authors contributed to the manuscript and approved the final version, and all authors agree to be accountable for the work.

## Conflict of Interest Statement

The authors declare that the research was conducted in the absence of any commercial or financial relationships that could be construed as a potential conflict of interest.
